# Evaluating the Effect a of Handout on Community Pharmacists’ Opioid Safety Counseling

**DOI:** 10.3390/pharmacy9010049

**Published:** 2021-02-28

**Authors:** Tanvee Thakur, Betty Chewning

**Affiliations:** Social and Administrative Sciences Division, School of Pharmacy, University of Wisconsin Madison, Madison, WI 53705, USA; betty.chewning@wisc.edu

**Keywords:** opioid, pharmacist, patient education, counseling, handout, safety

## Abstract

Community pharmacists are the most accessible healthcare professionals to counsel patients about opioid risks and safety. Resources such as handouts are needed to improve pharmacists’ self-efficacy about opioid safety counseling. This study aims to understand the effects and usefulness of handouts in opioid risk and safety counseling in community pharmacists. Three community pharmacies participated in this study for three weeks, where five pharmacists participated in completing a survey about the process and integration of a handout in opioid medication consultation. Pharmacists filled the survey after counseling patients for opioid medication/s. Field observations were conducted at one of these sites as well. A total of 57 consults were recorded via surveys in these pharmacies. Only using the handout to guide the conversation was rated much less useful than integrating the handout and showing it to patient in the consult (*β* = 0.94, adjusted *R*^2^ = 0.29, *p* < 0.00001). Satisfaction about the patient education provided increased if opioid risks and safety topics were discussed (*β* = 0.7, adjusted *R*^2^ = 0.32, *p* = 0.00015). Patients seemed engaged in the consults, which was evident from the head nods, questions asked, and attention toward the pharmacist. Effective communication with patients or patient caregivers about opioid safety can be accomplished by using and modeling use of this handout and by providing structured instructions to use this handout optimally.

## 1. Introduction

Community pharmacists are the most accessible healthcare professionals from whom patients can receive medication information [1]. Given this role, pharmacists have a professional responsibility to inform patients about the purpose, dose, side effects, risks, and safe use of medications and to optimize patient’s medication use and safety [2]. This information is all the more important for people who are prescribed medications, such as opioids, that are associated with risks of inappropriate use. 

Patients given opioids report they want community pharmacists to share information about opioid medication use and safety when they pick up their medication at the community pharmacy [3,4]. On the other hand, pharmacists have been slow to adopt their roles as opioid risk and safety educators [3]. There is an apparent conflict between patients’ desire for transparent information about opioids versus pharmacists’ reluctance to fill that gap. In response to the question of why pharmacists do not give patients more information about their opioids, several exploratory studies have identified pharmacists’ lack of resources, training, confidence, and time as barriers to discussing the risks of opioid medications with patients [3,5,6,7]. To reduce this gap, resources such as handouts (written educational leaflets for patient education) are needed to improve pharmacists’ self-efficacy and patient education about opioid risk and safety education [3]. To support a cognitive service for patients who are prescribed opioids as well as other medications, there is a need to systematically identify training approaches that are both scalable and effective [8]. Training may include simple instructions on how to use a handout and use it effectively. Using participatory research designs that involve pharmacists in developing instructions and training has proven effective in empirical literature [9,10]. Using a participatory research design process helps generate stakeholder-friendly training and interventions that make the resource use easier and feasible.

This study sought to evaluate the integration of a handout for opioid consults in community pharmacies. Specifically, this study explores the effects and usefulness of an opioid handout in opioid risk and safety counseling from the pharmacist’s perspective.

## 2. Methods

**Figure 1:**
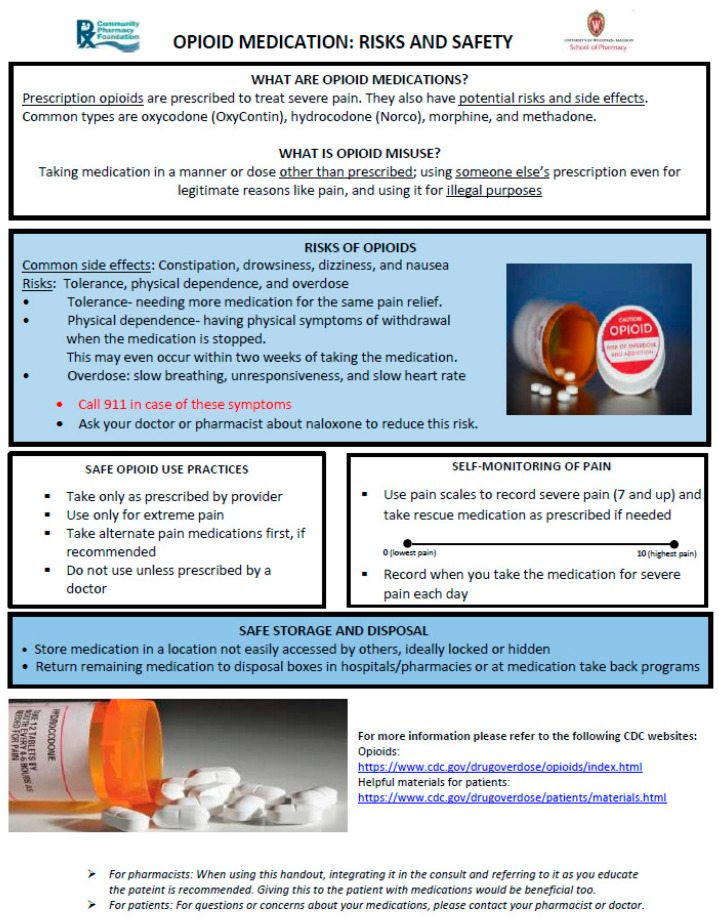
Opioid safety handout.

To accomplish the above objectives, the research team refined an opioid safety handout with pharmacist input (Figure 1) and integrated it into community pharmacy consultations. These consults were evaluated through rapid-cycle evaluations [11,12].

### 2.1. Study Design

The handout was developed and refined by the authors using an iterative process that involved interviewing community pharmacists to understand their perceptions and get their opinions on the content and format of the handout. This process is described elsewhere [13]. The handout was evaluated and piloted in three community pharmacies to assess the ease, technique, and impact of their implementation in consults. Participating community pharmacists counseled patients on the purpose, dose, side effects, risks, and safety practices for opioid medication that the patient was prescribed. Pharmacists in these pharmacies were asked to complete a short two-minute survey containing three questions about the topics they covered in each opioid consult for a week and what impact, if any, the handout had on their satisfaction and comfort about initiating and discussing opioid risks (see Appendix A for questionnaire). This questionnaire was adapted from the questionnaire used in another similar study [14]. Consults using this handout and pharmacy workflow were also observed at one community pharmacy site to identify potential barriers and facilitators for integrating the handout into practice. Pharmacists recorded their consults with patients visiting the participating pharmacy with a prescription for an opioid medication. Consults that involved participating pharmacists consulting patients on opioid medications were included in the study.

### 2.2. Sampling

Rapid evaluation of the pharmacist’s use of the handout during opioid consultations was conducted in three community pharmacies that were part of a stratified convenience sample. Pharmacies were chosen with varied locations, patient populations, and busyness of the pharmacy. A key criterion for including the pharmacies was that they were expected to dispense opioid prescriptions daily for children and/or adults. Participating pharmacies each received $200 for participating in this three-week study.

### 2.3. Analysis

The pharmacist self-reports of their handout use were analyzed primarily with descriptive statistics to identify how they used the handout, which topics they covered, barriers to using the handout, perceived patient responses, and the pharmacist’s comfort with the opioid risk portion of the consult. Bivariate regression analysis was conducted between topics covered and perceived patient responses. The statistical data analyses were conducted using R version 3.4.3 (2017, Vienna, Austria). Site observations conducted in the pharmacy were documented as field notes as well. 

## 3. Results

The study was conducted in three community pharmacies for three weeks. Two of these pharmacies were rural independent pharmacies and one was an urban chain pharmacy in the state of Wisconsin. A total of 57 consults were recorded via surveys in these pharmacies. Pharmacists were asked to use the handout in the way they felt was most effective in the consults. The handout used in this study can be found in Figure 1. A total of five pharmacists participated in the study. Three were female and two were male. The average number of years of practice for these pharmacists was 5 ± 2 years. 

### 3.1. Field Observations

Two field observations of opioid medication consults were conducted at the urban community pharmacy site across 15 h over four days. Observations were conducted by a trained fourth-year professional pharmacy student using an observation protocol. Based on the field notes and observations, pharmacists themselves referred to the handout during an opioid consult. Patients seemed engaged in the observed consults, which was evident from the head nods, questions asked, and attention toward the pharmacist. Pharmacists completed their surveys and immediately after the consult reported whether and how they integrated the handout into their patient opioid conversations.

### 3.2. Surveys 

**Figure 2:**
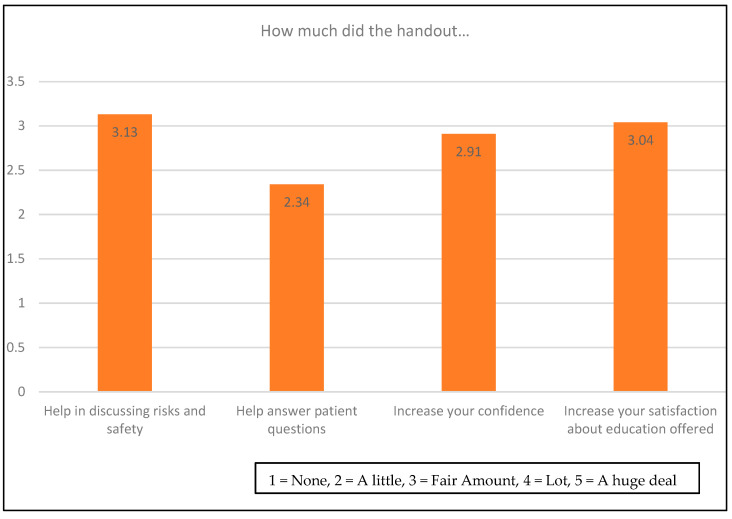
Effectiveness of the handout on a scale from 1 to 5.

Out of 57 consults, pharmacists integrated the handout into the consult and showed it to the patients in 21 consults. The pharmacists reported that the handout was not integrated into the consult but used as a guide in 34 consults. Two questionnaires did not report use of the handout. Average responses of pharmacists on a five-point scale about the use and effectiveness of the handout are presented in Figure 2. Pharmacists rated the handout as less useful when they used the handout to help guide the conversation rather than when integrating the handout and showing it to a patient in the consult (*β* = 0.94, adj. *R*^2^ = 0.29, *p* < 0.00001). The survey asked pharmacists about their satisfaction and comfort about the consultation when using the handout. If pharmacists discussed opioid risk and safety topics, they were more satisfied with education they provided to the patients (*β* = 0.7, adj. *R*^2^ = 0.32, *p* = 0.00015). Topics covered by pharmacists in the consults are displayed in Table 1.

## 4. Discussion

This study suggests that pharmacists viewed a simple handout as facilitating a fair amount of their communication with patients about opioid risks and safety. Similarly, pharmacists’ comfort and satisfaction were associated with their using the handout in the consult and with answering patient questions. Pharmacists who integrated the handout in the consult and used it to guide the conversation viewed the handout as more effective than just using it as a guide alone and not integrating it in the consult. During the two observed opioid consultations, patients also seemed to be involved in the consults that integrated the handout. The handout, which provides information to the patients in written format, is of paramount importance as a document as the patient can consult it any time whereas oral information can be forgotten or not all understood during counseling.

Pharmacists usually report opioid consultation barriers such as low self-efficacy and lack of sufficient resources to facilitate opioid risk and safety discussion with patients [5,6]. In other research, a participatory approach involving stakeholders helped enhance quality and outcomes of other interventions [9,10]. Mirroring this approach, this study’s handout had been refined through earlier pharmacist feedback. Using rapid evaluation feedback, this study explored implementation approaches and perceived usefulness of the handout in practice. Pharmacists counseled patients on dependency and overdose risks as well as on safe storage and disposal of opioids in more than one third of the consults. Pharmacists reported the handout to be more useful when they showed it to a patient during the consult than when just using it to help guide the conversation and not showing it to the patient during the consult. It is suggested that this approach should be tested to confirm effectiveness in different settings and should be promoted among healthcare professionals who use or plan to use a handout to educate patients about opioids. Pharmacists have expressed the need for more resources such as handouts and structured education on communicating with patients about opioids [3,14,15]. In this study, pharmacists used the handout as they preferred and documented characteristics and outcomes of its use after each encounter. This helped identify different approaches for using the handout to cover topics about risks and safety, with pharmacists suggesting some approaches were associated with higher reported pharmacist satisfaction and perceived effectiveness of the patient education. This study is the first of its kind to test approaches to include an opioid safety handout in counseling in community pharmacy setting. Another study conducted in an urban clinic, outpatient pharmacy reported similar findings where pharmacists reported an improvement in comfort and satisfaction with counseling patients on opioid safety after undergoing training about communication techniques and on including the handout in the consult [14]. This points to the suitability and efficacy of using opioid safety handouts across various pharmacy settings.

This study helps to inform a gap in research regarding pharmacists’ expression of a need for resources and training to facilitate their opioid risk and safety communications [3]. This intervention involved a sensitive topic due to the stigma around opioid risks [16]. Only providing pharmacists with resources, in this case the handout, is not enough. Pharmacists have also expressed a need for training about how to use or implement resources to facilitate opioid risks and safety communication. This study suggests the value of participatory approaches to assess the use of newly developed resources to maximize their effect for both healthcare professionals and patients. As a first step toward an agenda of intervention research, pharmacists themselves identified how best to implement the handout into their opioid consultations with patients. By doing so, in the process, they provided models for others. This handout is made available for public use by the Community Pharmacy Foundation and can be accessed on https://communitypharmacyfoundation.org/resources/grant_docs/CPFGrantDoc_22078.pdf (accessed on 15 January 2021) [17].

### Limitations

This pilot study has limitations with only 57 encounters with five pharmacists in three different community pharmacies. This study enrolled both rural and urban sites in Wisconsin, but the small number of sites involved limits the inferences and generalizability of this study to other pharmacy sites. There is a need to test this handout using a participatory design at other sites around the country to validate results from this study.

## 5. Conclusions

The handout evaluated in this study was valued by the pharmacists who tested it in their opioid consultations. It was reported to be effective in communicating sensitive topics including dependence, overdose, misuse and safe use, and storage of opioid medications to patients in community pharmacy settings. There is a need to replicate this demonstration and evaluation study in other sites, settings, and health professionals to facilitate conversations about opioid risks and safety.

## Figures and Tables

**Table 1:** Topics discussed during consults.

Topics	Frequency
Dose of medication	47
Common side effects	45
Safe storage	22
Dependency risks	20
Overdose risks	19
Safe disposal	17
Pain self-monitoring	11
Offered naloxone	6
